# Cardiovascular Disease and Cardiovascular Disease Risk in HIV-Positive Populations in the Asian Region

**DOI:** 10.2174/1874613601711010052

**Published:** 2017-08-21

**Authors:** Rimke Bijker, Jun Yong Choi, Rossana Ditangco, Sasisopin Kiertiburanakul, Man Po Lee, Sarawut Siwamogsatham, Sanjay Pujari, Jeremy Ross, Chi-yuen Wong, Wing-Wai Wong, Evy Yunihastuti, Matthew Law

**Affiliations:** 1 The Kirby Institute, UNSW, Sydney, NSW 2052, Australia; 2 Department of Internal Medicine and AIDS Research Institute, Yonsei University College of Medicine, Seoul, Korea; 3 Research Institute for Tropical Medicine, Manila, Philippines; 4 Faculty of Medicine Ramathibodi Hospital, Mahidol University, Bangkok, Thailand; 5 Queen Elizabeth Hospital, Kowloon, Hong Kong; 6 King Chulalongkorn Memorial Hospital, Bangkok, Thailand; 7 Faculty of Medicine, Chulalongkorn University, Bangkok, Thailand; 8 Institute of Infectious Diseases, Pune, India; 9 TREAT Asia, Bangkok, Thailand; 10 Taipei Veterans General Hospital, Taipei, Taiwan; 11 Cipto Mangunkusumo General Hospital, Jakarta, Indonesia

**Keywords:** HIV, Cardiovascular disease, Cardiovascular risk factors, Asia

## Abstract

**Introduction:**

Cardiovascular diseases (CVD) are becoming more prevalent in HIV-infected populations as they age largely due to improved treatment outcomes. Assessment of CVD risk and CVD risk factors in HIV-positive populations has focused on high income settings, while there are limited studies evaluating CVD in HIV-positive populations in the Asian region.

**Materials and Methods:**

We provided an overview of the prevalence and incidence of CVD and its risk factors in adult HIV-positive populations, and of the strategies currently in place for CVD management in the Asian region.

**Results:**

Studies from the Asian region showed that CVD and CVD risk factors, such as dyslipidaemia, elevated blood glucose, obesity and smoking, are highly prevalent in HIV-positive populations. A number of studies suggested that HIV infection and antiretroviral therapy may contribute to increased CVD risk. National HIV treatment guidelines provide some directions regarding CVD risk prevention and management in the HIV-infected population, however, they are limited in number and scope.

**Conclusion:**

Development and consolidation of guidelines for integrated CVD and HIV care are essential to control the burden of CVD in HIV-positive populations. To inform guidelines, policies and practice in the Asian region, research should focus on exploring appropriate CVD risk screening strategies and estimating current and future CVD mortality and morbidity rates.

## 1 INTRODUCTION

Due to largely improved antiretroviral therapy (ART) outcomes, HIV positive individuals are growing older and as a result chronic diseases associated with aging, including cardiovascular diseases (CVD) and cancer, are becoming more prevalent in the HIV-infected population [1]. A recent review of data from high income countries estimated that HIV populations have around a twofold increased risk of experiencing CVD events compared to the general population [2].

 Similar to risk factors in the general population, CVD in the HIV-infected population can be caused by non-modifiable risk factors including older age and family history of CVD, and modifiable risk factors such as medical conditions (e.g. hypertension, diabetes, dyslipidaemia) and behavioural factors (e.g. smoking and diet). Furthermore, the increased risk of CVD might also be attributable to risk factors specific to the HIV-positive population, such as compromised immunity [3, 4], immune activation, HIV associated inflammation [5], and receiving ART [6, 7].

Assessment of CVD risk and CVD risk factors in HIV populations has focused on high income settings, while there are limited studies evaluating CVD in HIV populations in the Asian region, where income levels greatly differ across countries [8]. This review aims to provide insight on the prevalence and incidence of CVD and its risk factors in adult HIV populations in the Asian region.

 We defined CVD as stroke, myocardial infarction and coronary heart disease (CHD) and we considered blood lipid levels, diabetes and blood glucose levels, abdominal obesity, hypertension and smoking as CVD risk factors. We report research findings from the last 10 years of studies conducted in China, India, Indonesia, Japan, Malaysia, South Korea, Taiwan, Thailand, Cambodia, Myanmar, the Philippines and Vietnam.

 When required, differences were reported between HIV negative, ART naïve, and ART experienced populations. In addition, we provide an overview of the strategies currently in place for CVD management in HIV-populations in the Asian region. We conclude with future directions for practice and research.

## 2 CARDIOVASCULAR DISEASE AND CARDIAC FUNCTIONING

Few studies have evaluated cardiovascular health in HIV-positive populations in the Asian region. Table **1** provides an overview of studies evaluating CVD events and surrogate markers of cardiovascular dysfunction. One recent Taiwanese study assessed the incidence of stroke in patients with HIV compared with non-infected individuals from the Taiwan National Health Insurance Research Database [9]. The study showed that stroke occurred more frequently in those with HIV (0.96% *vs.* 0.24%).

 In fact, independent of other traditional CVD risk factors, HIV infection was associated with a 2.75 fold increased risk (95% Confidence Interval [CI] 1.17-6.44) of having a stroke [9]. Further evidence of CVD risk stems from studies investigating surrogate measures of CVD, such as carotid artery plaque and carotid intima-media thickness. A study from South Korea found that 23.4% of HIV patients receiving ART had carotid artery plaque and that ART use was significantly associated with the existence of carotid artery plaque [10]. In a small Indian study population, no significant difference was found in carotid intima-media thickness between ART experienced and ART naïve patients [11].

Several studies in the Asian region have used two-dimensional echocardiogram to assess cardiac function in HIV-positive populations. In India, echocardiographic abnormalities were identified in 67.0% of HIV-infected patients [12]. Diastolic dysfunction (DD) was the most common abnormality occurring in 48.2% of patients, followed by left ventricular systolic dysfunction occurring in 17.6% of patients.

 Similar proportions of DD were found in Chinese patients on ART, which was substantially lower compared to proportions of DD (20%) detected in HIV negative individuals [13]. A Chinese longitudinal study found that cardiac abnormalities, including left ventricular systolic dysfunction, DD, increased left ventricular mass index and pulmonary arterial hypertension, were more prevalent 48 weeks after ART initiation, although this finding was only significant for DD [14]. This study also showed HIV infection to be independently associated with DD and increased left ventricular mass index.

## 3 ESTIMATES OF CARDIOVASCULAR DISEASE RISK

Additional information on CVD risk among HIV-positive populations is provided by studies that estimate CVD risk using various risk assessment systems, such as the Rama-EGAT risk score, the Framingham risk score (FRS) and the D:A:D risk score. Risk scores of ≥10% are commonly considered as having high risk of developing CVD in the future. One study investigated various CVD risk outcomes in a multi-country analysis and showed that high 5-year risk of CVD, CHD and myocardial infarction was prevalent in 16, 11 and 6% of the treated population, respectively [15].

 In South Korea, 10-year CVD risk was compared among HIV positive and negative patients using the FRS [16]. High CVD risk was more common in HIV positive than in HIV negative individuals (29.3 *vs.* 19.5%), although this difference was not significant.

 Using the Rama-EGAT risk score, one study estimated that 11% of the HIV patients from a clinic in Bangkok had a high 10-year risk of CVD events [17]. Another Thai study estimated the 10-year risk for Coronary Heart Disease (CHD) with each of the scoring systems and found that the prevalence of high 10-year CHD risk was 0.8%, 2.1% and 9.9% as calculated by the D:A:D, Rama-EGAT and FRS, respectively [18].

## 4 RISK FACTORS OF CARDIOVASCULAR DISEASE

Table **2** provides an overview of the studies on modifiable risk factors of CVD and HIV.

### 4.1 Blood Lipid Profiles

Blood lipid profiles are generally considered abnormal when there are high levels of triglycerides (≥150 mg/dL), total cholesterol (TC ≥200 mg/dL), low-density lipoprotein cholesterol (LDL ≥130 mg/dL) and/or low levels of HDL (<40 mg/dL).

Abnormalities in one or more of these blood lipids are commonly referred to as dyslipidaemia. Several studies from the Asian region showed that dyslipidaemia was less prevalent in patients who were first presented with HIV compared to their HIV negative counterparts [19, 20]. Cholesterol levels were on average lower in HIV positive patients [19-22], whereas findings were inconsistent regarding triglyceride levels [19-21].

Treatment with ART appears to have an unfavourable effect on blood lipid levels. In Malaysia, dyslipidaemia was reported in 82% of patients on ART [23]. Other evidence from the Asian region indicated abnormal levels of triglycerides, TC, LDL and HDL in 7-39%, 3-8%, 2-17% and 19-86% of ART naïve patients, respectively [19-22, 24-26], compared to 39-54%, 26-54%, 22-48% and 14-68% of ART experienced patients, respectively [15, 19, 20, 23-27].

 Moreover, a Thai study showed that the prevalence of abnormal lipid levels was even higher in those on PI-containing ART regimens [28]. Studies from high, upper-middle and lower-middle income groups all confirmed significant associations between ART use and unfavourable lipid levels [16, 19, 25, 26, 29, 30].

### 4.2 Diabetes and Abnormal Blood Glucose Levels

Information regarding glucose levels has frequently been described in studies from India. In a study comparing glucose levels among ART experienced patients, ART naïve patients and HIV negative individuals, hyperglycaemia (fasting plasma glucose ≥100 mg/dL) was observed in 34, 21 and 31% of the participants, respectively [19]. In contrast, in a study evaluating ART naïve and experienced patients, no significant difference in hyperglycaemia prevalence was found between the two patient groups [24]. Interestingly, one study showed a significant increase in blood glucose levels in HIV patients after they started ART [29]. In fact, after six months of ART, 4.2% of patients were newly diagnosed with impaired fasting glucose (fasting plasma glucose 100-125 mg/dL), while an additional 2.1% of patients developed diabetes (fasting plasma glucose >125 mg/dL). A cohort study evaluated blood glucose levels in tuberculosis co-infected HIV patients [30]. Twelve months after ART initiation, elevated blood glucose levels (>110 mg/dL) had developed in 11% of patients. However, compared to before ART initiation there was no significant difference in the prevalence of abnormal blood glucose levels, since abnormalities did not persist in all baseline cases [30]. Two studies evaluated insulin resistance and found it more frequent in those on ART than in those who had not yet started treatment, albeit not significantly [19, 31].

Additional information on diabetes and blood glucose levels was available from other Asian countries. In China, blood glucose levels were higher in HIV positive than HIV negative men [22]. Furthermore, 20% of Chinese ART-naïve patients had elevated blood glucose levels, of whom 11% met the criteria for diabetes [32]. Other findings from China showed that diabetes and impaired fasting glucose developed relatively frequently in patients after they initiated ART (incidence of 2.62 and 35.64 per 100 person-years, respectively) [33]. Substantially, lower diabetes incidence rates were found in ART experienced patients in Thailand (5.0 per 1000 person-years) [34] and in high-income country Taiwan (13.1 per 1000 person-years) [35]. In Thailand, the diabetes prevalence was 5.4% in a study population 10 years after HIV diagnosis [27] and (depending on the type of regimen) ranged from 1.7-6.6% in another ART treated study population [28], while impaired fasting glucose was seen in 28% of a non-diabetic population of whom the majority was on ART [36]. In Malaysian patients on ART, the prevalence of diabetes and hyperglycaemia was 13 and 38%, respectively [23]. A lower prevalence of hyperglycaemia was reported among HIV infected patients assessed in Thailand, with the proportion in ART experienced patients being higher than in ART naïve patients (24 *vs.* 12%, p <0.05) [25]. Lastly, an analysis on the wider Asian region showed a mean diabetes prevalence of 8.3% [15].

### 4.3 Hypertension

There is inconclusive evidence from the Asian region regarding hypertension and differences across HIV or ART status. For example, no significant differences were found between HIV negative and positive men in China [22]. In addition, ART naïve patients in India had a relatively low prevalence of hypertension (12%) compared to HIV negative individuals (36%) and ART experienced patients (17%, p=0.002) [19]. Other findings from India indicate no difference with regard to hypertension between ART naïve and experienced patients [24], while a cohort study showed an increase in average systolic and diastolic blood pressure after 6 months on ART, with 10% of HIV patients having developed hypertension [29]. A Thai study, however, reported no significant difference in the prevalence of hypertension between ART naïve and ART experienced patients (22 *vs.* 28%) [25]. In Malaysia, hypertension was reported in almost half of HIV patients on ART [37]. Finally, a multi-country study showed that around 20% of ART experienced individuals had hypertension [15].

### 4.4 Metabolic Syndrome

Based on studies from India, Thailand and Taiwan, the prevalence of metabolic syndrome, a set of clinical parameters associated with the development of heart disease and other health problems such as diabetes, varied from 19-43% for HIV patients who were on ART [24, 25, 31, 38]. Findings suggest that ART has a negative effect on metabolic functioning. This is illustrated by two studies that reported a significantly higher prevalence of metabolic syndrome in ART experienced than ART naïve patients [25, 31] and by a longitudinal study that described new onset metabolic syndrome in 25% of study participants 6 months after ART initiation [29].

### 4.5 Abdominal Obesity

Abdominal obesity is a well-known contributor to hypertension, diabetes and metabolic syndrome. In ART experienced HIV patients in Thailand abdominal obesity was prevalent in 39% [25], which is similar to findings from an Indian study [19]. In both studies, the prevalence in patients on ART was comparable to ART naïve peers. Interestingly, findings from an Indian cohort study suggest that ART might eventually negatively affect healthy body mass, with study participants having increased BMI and skinfold thickness after 6 months of ART [29]. Likewise, waist circumference and waist-to-hip ratio were significantly higher in ART experienced compared to ART naïve individuals [28].

### 4.6 Tobacco Use

Across the Asian region, the proportion of current smokers ranged from 23-62% [15, 22, 39-41] and that of former smoker from 10-20% [15, 40]. One study specifically compared smoking among Asian populations on ART and found that compared to Thailand, the prevalence of smoking was lower in India and the Philippines, while it was higher in Vietnam, Indonesia, Hong Kong, Taiwan, Malaysia, Japan, South Korea and Singapore [15]. In other studies, the prevalence of current smoking was notably higher in men [40, 42]. Furthermore, when comparing across HIV status, in India those with HIV were more likely to smoke (p <0.05) [42], whereas in Chinese men who had sex with men there were no significant differences between those with or without HIV [22].

## 5 MANAGEMENT OF CVD IN HIV POPULATIONS

Since HIV populations are at increased CVD risk, screening approaches designed for the general population may underestimate individual CVD risk. Hence, it is recommended that clinicians consider using HIV specific risk scoring systems or take into account that HIV infection is an additional risk factor and provide CVD preventive measures accordingly. As shown by a meta-analysis of observational studies, increased CVD risk might be attributable to specific antiretroviral drugs, such as abacavir, indinavir and lopinavir [43]. Newer generations of drugs could diminish CVD risk [44], however, these drugs might not be available in low-resource settings. It is of particular importance that clinicians carefully select ART regimens, thereby limiting adverse events that increase CVD risk. Moreover, health systems should plan for and ensure access to the most appropriate ART.

Globally, there are limited specific guidance and recommendations on the joint management and prevention of CVD in HIV patients. However, guidelines for HIV treatment do offer some support on the management of CVD and associated risks in HIV-infected patients. For example, in limited resource settings, WHO HIV treatment guidelines recommend the use of statins for people with a 10-year CVD risk exceeding 30%, and caution the use of boosted protease inhibitors with lovastatin and simvastatin as drug interactions may lead to increased risk of developing serious adverse effects [45]. Although national HIV treatment guidelines from the Asian region generally provide recommendations with regard to screening and monitoring of patients prior to ART initiation and afterwards (Table **3**), the current routinely collected patient data in some countries might be insufficient to estimate individual CVD risk and provide preventive treatment accordingly. Furthermore, not all guidelines provide information with regard to possible interactions between ART and other drugs (i.e. antiplatelet or statin) that might prevent or treat CVD [46-48].

## CONCLUSION AND FUTURE DIRECTIONS

The limited findings available from this review suggest that HIV-positive populations in Asia are prone to developing CVD. While compared to HIV-negative individuals, blood lipid levels tend to be lower in newly diagnosed HIV patients, an unfavourable rise in lipid levels is common after ART initiation. Furthermore, compared to HIV-negative individuals, smoking is highly prevalent in those with HIV, especially in men. Additionally, a substantial proportion of HIV populations appear to be affected by cardiac abnormalities and other CVD risk factors such as high glucose levels, metabolic syndrome and abdominal obesity. Caution is warranted when interpreting these findings since there may be important sociodemographic differences between Asian countries, such as those related to country income level. Furthermore, comparing the findings between countries is difficult due to differences across studies, such as study definitions, measures to define CVD, study populations (e.g. size and selection criteria) and study limitations, indicating that more research is warranted on CVD and CVD risk factors in Asia. Nonetheless, these data suggest that as the HIV population ages, CVD will become an increasing source of morbidity and mortality in the Asian region.

English versions of national guidelines for HIV treatment include limited recommendations on CVD risk assessment and management. Although guidelines published in national language may be more specific, it is unclear to what extent these are implemented. Clinicians caring for HIV-infected patients should have guidance on assessing and managing CVD and associated risks, and optimal treatment options and drug-drug interactions readily available. Furthermore, in the context of the strained health systems and competing health priorities of low-to-middle income countries, it might be challenging to ensure access to CVD services for HIV patients who are at increased CVD risk, especially for key affected populations. To overcome these issues, there is an urgent need for guidelines that integrate CVD risk prevention and management into HIV care.

Optimal prevention and management are vital in dealing with the rising burden of CVD in HIV-positive populations. This review illustrates that there is limited research on CVD in HIV-positive populations in the Asian region. In particular, there is a need for more large-scale studies which compare absolute CVD events with estimated CVD risk in HIV positive and negative populations. In addition, future research should assess the level of implementation of guidelines regarding CVD risk assessment and management. Lastly, further work is warranted to explore effective CVD risk screening approaches that are feasible in the Asian region, where resources may be limited.

## Figures and Tables

**Table 1:** Summary of studies assessing the prevalence or incidence of CVD, cardiac abnormalities and CVD risk.

**Study (refs)**	**Country, income group**	**Study design**	**Population** **N (% male), *mean age (SD)***	**Outcome**	**Findings**
**Toh, 2014 [****9****]**	Taiwan, high income	Cross-sectional	622 HIV+ and 1244 HIV- (unsp.) *34.5 years*	Stroke	Incidence of stroke in HIV+ *vs.* HIV- -0.96% *vs.* 0.24% (p=0.0678) Differences between HIV+ and HIV-Those with HIV more frequently had stroke (OR 2.75, 95% CI 1.17-6.44, p=0.0199) *^#^*
**Jeong, 2013 [****10****]**	South Korea, high income	Cross-sectional	145 HIV+ (96%) *40.9 years (10.8)*	Surrogate CVD measure	Prevalence of carotid artery plaque -23.4% Differences between on ART and ART naïve -Those on ART more frequently had carotid artery plaque (p<0.05) *^#^*
**Suparna, 2013 [****11****]**	India, lower-middle income	Cross-sectional	42 HIV+ (67%): 26 on ART, *28.6 years (7.0)*; and 16 ART naïve, *34.1 years (6.9)*	Surrogate CVD measure	Differences between on ART and ART naïve -No significant difference in carotid intima media thickness (p=0.46)
**Jain, 2014 [****12****]**	India, lower-middle income	Cross-sectional	100 HIV+ (78%), *37.3 years (8.7) ^^^*	Cardiac abnormalities	Prevalence of cardiac abnormalities*^¥^* -Cumulative cardiac abnormalities: 67.0% -DD: 42.8%*^±^* -Left ventricular systolic dysfunction: 17.6%*°*
**Luo, 2010 [****13****]**	China, upper-middle income	Cross-sectional	42 AIDS (60%), 40.6 years (9.3); 42 HIV+ (60%), 38.6 years (6.5); 30 HIV- (60%), 41.5 years (10.5)	Cardiac abnormalities	Prevalence cardiac abnormalities: AIDS *vs.* HIV+ *vs.* HIV-*^¥^* -DD: 48 *vs.* 43 *vs.* 20% (p<0.05 for AIDS *vs.* HIV- and AIDS *vs.* HIV+) *^±^* Differences between HIV+ and HIV- *^#^* - Those with HIV more frequently had DD (OR 17.00, 95%CI 3.33-86.9, p <0.001)
**Luo, 2014 [****14****]**	China, upper-middle income	Longitudinal case-control	325 starting ART (73%), *38.2 years (10.1)*; and 97 HIV- (80%), *37.8 years (11.0)*	Cardiac abnormalities	Prevalence cardiac normalities: on ART (after 48 weeks) *vs.* ART naïve *vs.* HIV-*^¥^* -DD: 23.3 *vs.* 16.5 *vs.* 7.2%, (p=0.027 for ART naïve *vs.* HIV-) -Left ventricular systolic dysfunction: 9.8 *vs.* 7.3 *vs.* 2.1% (p=ns) *°* -Pulmonary arterial hypertension: 6.9 *vs.* 2.7 *vs.* 7.3% (p=ns) -Increased left ventricular mass: 15.6 *vs.* 11.1 *vs.* 5.1% (p=ns) *~* Differences between HIV+ and HIV- *^#^* -Those with HIV more frequently had increased left ventricular mass (OR 3.76, 95%CI 1.13-12.53, p=0.031) and DD (OR 3.65 95%CI 1.34-9.98 p=0.012)
**Do, 2016 [****15****]**	Asia (Thailand, Vietnam, Indonesia, India, Hong Kong, Philippines, Taiwan, Malaysia, Japan, South Korea, Singapore, China), N/A	Cross-sectional	1496 on ART (68%), 44.0 years (38.5-50.4)**		Prevalence of high (≥10%) 5-year risk of CVD, CHD, and MI -CVD: 15.5% -CHD: 11.1% -MI: 5.6%
**Kim, 2013 [****16****]**	South Korea, high income	Cross-sectional case-control	116 HIV+(97%) and 226 HIV- (95%), *41.9 years (8.9)*	10-year CVD risk	Prevalence of 10 year-CVD risk in HIV+ *vs.* HIV- -<10% risk: 70.7 *vs.* 80.5%; -10-15% risk: 14.7 *vs.* 10.6%; -16-20% risk: 5.2 *vs.* 6.2%; ->20% risk: 1.7 *vs.* 2.7% -(p=0.78)
**Suppadungsuk, 2013 [****17****]**	Thailand, upper-middle income	Cross-sectional	109 (67%), *47.3 years (9.7)*	High 10-year CVD risk*	Prevalence of high (≥10%) 10 year-CVD risk -11% (Rama-EGAT)
**Edwards-Jackson, 2011 [****18****]**	Thailand, upper-middle income	Cross-sectional	785 (55%), *41.0 years (7.7)*	High 10 year CHD risk *	Prevalence of high (≥10%) 10 year-CHD risk -0.8% (D:A:D) -2.1% (Rama-EGAT) -9.9% (FRS)

Abbreviations: N=number; SD=standard deviation; unsp.=unspecified; OR=odds ratio; CI=confidence interval; p=probability; CHD=coronary heart disease; ART=antiretroviral therapy; CVD=cardiovascular disease; HIV+/-=HIV positive/negative individuals; *vs.*=versus; DD=diastolic dysfunction; MI=myocardial infarction. * >10% risk; ^#^ findings from multivariate analysis; ^^^ study included patients aged >13 year; ^¥^ measured by two-dimensional transthoracic echocardiography; ^±^ defined according to pulse wave Doppler parameters for mitral inflow; ° defined by left ventricular ejection fraction <45%; ~ ≥ 110 g/m2 after body surface area indexation.

**Table 2:** Summary of studies assessing the prevalence or incidence of CVD risk factors.

**Study (refs)**	**Country, income group**	**Study design**	**Population** **N (% male), *mean age (SD)***	**Outcome(s)**	**Findings**
**Carey, 2013 [****19****]**	India, lower-middle income	Cross-sectional	59 on ART (58%), *39.2 years (9.1)*; 66 ART naïve (34%), *33.1 years (6.1);* and 75 HIV- (69%), *37.7 years (8.7)*	Lipid levels, diabetes, hypertension, obesity	Prevalence of medical risk factors: ART *vs.* ART naïve *vs.* HIV- -Abdominal obesity: 35.0 *vs.* 36.5 *vs.* 46.7% (p=ns) -Hypertension: 17.3 *vs.* 12.1 *vs.* 36.0% (p=0.002) -Hyperglycaemia: 34.0 *vs.* 20.7 *vs.* 31.2% (p=ns) -Insulin resistance: 8.2 *vs.* 3.6 *vs.* 3.4%(p=ns) -High TC: 36.0 *vs.* 6.9 *vs.* 20.0% (p=0.001) -Low HDL: 54.0 *vs.* 86.2 *vs.* 78.4% (p<0.001) -High LDL: 34.0 *vs.* 17.2 *vs.* 35.4% (p=0.05) -High triglycerides: 48.0 *vs.* 17.2 *vs.* 20.0% (p<0.001) Differences between on ART and ART naïve*^^^* -Those on ART more frequently had high triglycerides (OR 5.2, 95%CI 1.3-20.3, p=0.019) -Those on ART less frequently had hypertension (OR 0.2, 95%CI 0.03-0.9, p=0.037), and high triglycerides (OR 8.0, 95%CI 1.9-32.2, p=0.003)
**Kalyanasundaram, 2012 [****20****]**	India, lower-middle income	Cross-sectional	145 on ART (47%); 146 ART naïve (51%); and 72 HIV- (54%), *33.9 years (7.2)*	Lipid levels	Prevalence of medical risk factors: ART *vs.* ART naïve *vs.* HIV- -High TC: 38.6 *vs.* 4.1 *vs.* 16.7%, p<0.05; -Low HDL: 24.8 *vs.* 73.3 *vs.* 40.3%, p<0.05; -High LDL: 31.0 *vs.* 6.2 *vs.* 15.3%, p<0.05; -High triglycerides: 42.1 *vs.* 6.8 *vs.* 20.8%, p<0.05
**Shen, 2015 [****21****]**	China, upper-middle income	Cross-sectional	1518 ART naïve (75%), 38 years (unsp.)*; and 347 HIV-(36%), 37 years (unsp.)*	Lipid levels	Prevalence of medical risk factors: HIV+ *vs.* HIV- -High TC: 8.4 *vs.* 28.2% (p<0.001) -Low HDL: 59.6 *vs.* 11.2% (p<0.001) -High LDL: 8.5 *vs.* 62.6% (p<0.001) -High triglycerides: 33.9 *vs.* 17.0% (p<0.001) Differences between HIV+ and HIV- *^^^* -Those with HIV less frequently had high TC (OR 0.29, 95%CI 0.21-0.41, p<0.001) and high LDL (OR 0.06, 95%CI 0.04-0.09, p<0.001) -Those with HIV more frequently had high triglycerides (OR 2.77 95%CI 2.04-3.75 p<0.001), and low HDL (OR 8.58 95%CI 5.27-13.96 p<0.001)
**Wang, 2016 [****22****]**	China, upper-middle income	Case Control	320 men who have sex with men of whom 100 HIV- (100%), 32.2 years (9.0); 110 ART naïve acute HIV ART (100%.), 31.5 years (8.6); 110 ART naïve chronic HIV infection (100%.), 33.8 years (6.2)	Lipid levels, hypertension, blood glucose levels, tobacco use	Prevalence of medical risk factors: HIV- *vs.* acute HIV *vs.* chronic HIV -High TC: 21.0 *vs.* 6.4 *vs.* 2.7% (p<0.05 HIV- *vs.* acute and *vs.* chronic) -Low HDL: 17.0 *vs.* 33.6 *vs.* 40.0% (p<0.05 HIV- *vs.* acute and *vs.* chronic) *^±^* -High LDL: 16.0 *vs.* 1.8 *vs.* 1.8% (p<0.05 HIV- *vs.* acute and *vs.* chronic) *^∞^* -High triglycerides: 23.0 *vs.* 15.5 *vs.* 20% (p=ns) -Dyslipidemia: 44.0 *vs.* 46.4 *vs.* 47.3% (p=ns) -Hypertension: 17.0 *vs.* 9.1 *vs.* 17.3% (p=ns) Prevalence of behavioural risk factors: HIV- *vs.* acute HIV *vs.* chronic HIV -Ever Smoking: 38.0 *vs.* 26.4 *vs.* 33.6% (p=ns) Differences between HIV- and acute HIV -Those with acute HIV had lower triglycerides, TC, HDL and LDL (p<0.05) Differences between HIV- and chronic HIV -Those with chronic HIV had lower triglycerides, TC, HDL and LDL (p<0.05) -Those with chronic HIV had higher blood glucose levels (p<0.05)
**Hejazi, 2013 [****23****]**	Malaysia, upper-middle income	Cross-sectional	2739 on ART (81%), *43.1 years (9.9)*	Lipid levels, diabetes	Prevalence medical risk factors -High TC: 54.2% -Low HDL: 28.7% -High LDL: 35.1% -High triglycerides: 59.1% -Dyslipidaemia: 82.3% -Hyperglycaemia: 38.2% -Diabetes mellitus: 12.9%
**Do, 2016 [****15****]**	Asia (Thailand, Vietnam, Indonesia, India, Hong Kong, Philippines, Taiwan, Malaysia, Japan, South Korea, Singapore, China	Cross-sectional	4274 HIV (71%), 40.7 (34.5-47.6)*** Data available medical risk factors: 1496 on ART (68%), 44.0 years (38.5-50.4)**	Lipid levels, diabetes, hypertension, tobacco use	Prevalence medical risk factors -Hypertension: 20.3% -Diabetes mellitus: 8.3% -High TC: 45.9%*~* -Low HDL: 21.5% -High triglycerides: 39.0%^€^ Prevalence behavioural risk factors -Current smoking: 23.2% -Former smoking: 20.3% Differences between countries -Compared the Thailand, the prevalence of smoking was significantly lower in India and the Philippines and higher in Vietnam, Indonesia, Hong Kong, Taiwan, Malaysia, Japan, South Korea and Singapore (p<0.001)
**Bajaj, 2013 [****24****]**	India, lower-middle income	Cross-sectional	70 HIV+ (71%), *age unsp*.: 47 on ART; 23 ART naïve	Metabolic syndrome and its components	Prevalence of medical risk factors: ART *vs.* ART naïve -Metabolic syndrome: 19.1 *vs.* 21.7% (p=ns) -Low HDL: 55.3 *vs.* 34.8% (p=ns) -High triglycerides: 44.7 *vs.* 39.1% (p=ns) -Hyperglycaemia: 27.7 *vs.* 30.4% (p=ns) *^¥^* -Hypertension: 14.9 *vs.* 8.7% (p=ns)
**Jantarapakde, 2014 [****25****]**	Thailand, upper-middle income	Cross-sectional	410 on ART (51%), *39 years (34-44)*; and 170 ART naïve (35%), *34 years(29-40) ^#^*	Metabolic syndrome and its components	Prevalence of medical risk factors: ART *vs.* ART naïve -Metabolic syndrome: 24.9 *vs.* 15.9 (p=0.018) -Hypertension: 27.8 *vs.* 22.4 (p=ns) -Low HDL: 40.0 *vs.* 70.6 (p<0.05) -High triglycerides: 47.8 *vs.* 25.3 (p<0.05) -Hyperglycaemia: 24.4 *vs.* 11.7 (p<0.05) -Abdominal obesity: 29.0 *vs.* 32.9% (p=ns)
**Indumati, 2014 [****26****]**	India, lower-middle income	Cross sectional	100 ART naïve (67%), 35.8 years (9.1); 100 on ART (60%), 36.0 years (9.0)	Lipid levels	Prevalence of medical risk factors: ART *vs.* ART naïve -High TC: 49 *vs.* 8% (<0.0001) -Low HDL: 68 *vs.* 81% (p=0.0366) -High LDL: 48 *vs.* 5% (p<0.0001) -High triglycerides: 48 *vs.* 27% (p=0.0024) Differences between on ART and ART naïve -Those on ART had higher TC (P<0.0001), LDL (P<0.0001), triglycerides (p=0.0045)
**Kiertiburanakul, 2012 [****27****]**	Thailand, upper-middle income	Cross-sectional	129 on ART (50%), *46.2 years (9.3)*	Lipid levels, diabetes	Prevalence of medical risk factors -Dyslipidaemia: 58.9% -High TC: 25.6% *~* -Low HDL: 14.0% *^±^* -High LDL: 21.7% *°* -High triglycerides: 50.4% -Diabetes: 5.4%
**Kim, 2013 [****16****]**	South Korea, high income	Cross-sectional case-control	116 on ART (97%); and 226 HIV- (95%), *41.9 years (8.9)*	Lipid levels, blood pressure	Differences after ART initiation) -After 6 months ART individuals had increased TC (p<0.001); HDL (p<0.001); LDL (p=0.007); and triglycerides (p<0.001) Differences between on ART and HIV- -Those on ART had lower TC (p=0.023), HDL (p=0.019), LDL (p<0.001), and BMI (p<0.001) - Those on ART had higher triglycerides (p<0.001), systolic BP (p<0.001), and diastolic BP (p<0.001)
**Homsanit, 2007 [****28****]**	Thailand, upper-middle income	Cross-sectional	126 naïve (28%), 33.6 years (6.5); 61 on PI-containing ART (57%), 38.9 years (6.7); 60 on other ART (45%), 37.0 years (8.4)	Lipid levels, diabetes, abdominal obesity	Prevalence of medical risk factors: ART naïve *vs.* PI containing ART *vs.* non-PI ART -High TC: 18.3 *vs.* 75.4 *vs.* 58.3% (p<0.001) -Low HDL: 42.9 *vs.* 31.2 *vs.* 20% (p=0.007) -High LDL: 15.1 *vs.* 59.1 *vs.* 41.7% (p<0.001) -High triglycerides: 8.2 *vs.* 75.4 *vs.* 48.3% (p<0.001) -Diabetes: 0 *vs.*. 6.6 *vs.* 1.7% (p=0.04) Differences between ART naiive and those on PI containing ART -Those in PI-containing ART had higher waist circumference (p=0.004) and waist-to-hip ratio (p<0.001) Differences between ART naiive and those on non-PI containing ART -Those in non-PI-containing ART had higher waist circumference (p=0.002) and waist-to-hip ratio (p<0.001)
**Gupta, 2011 [****29****]**	India, lower-middle income	Longitudinal	68 starting ART (84%), *35.9 years (9.4)*	Metabolic syndrome and its components	Incidence rate of medical risk factors after 6 months ART -Metabolic syndrome:25.0% -Hypertension: 10.3% -Impaired fasting glucose: 4.2% -Diabetes mellitus: 2.1% Differences after ART initiation -After 6 months ART, individuals had increased systolic BP, diastolic BP, BMI, skinfold thickness, blood glucose, TC, HDL and LDL
**Padmapriyadarsini, 2011 [****30****]**	India, lower-middle income	Longitudinal	168 TB co-infected (79%), on EFV: *35 years (6.9)*; on NVP: *37 years (7.7)*	Lipid levels, glucose levels	Incidence rate of medical risk factors after 12 months ART -High TC: 26% -Low HDL: 23% -High LDL: 0% -High triglycerides: 20% -Elevated blood glucose levels: 11% ^¥^ Differences after ART initiation -After 6 and 12 months of ART, individuals had increased cholesterol, HDL and LDL (p<0.001)
**Idiculla, 2011 [****31****]**	India, lower-middle income	Cross-sectional	30 on ART (77%), *41.6 years (10.9)*; and 30 ART naïve (70%), *40.6 years (9.5)*	Metabolic syndrome and its components	Prevalence of medical risk factors: ART *vs.* ART naïve -Metabolic syndrome: 43.3 *vs.* 10% (p=0.028); -Insulin resistance: 62.5 *vs.* 37.5% (p=ns)
**Shen, 2013 [****32****]**	China, upper-middle income	Cross-sectional	2006 ART naïve (76%) 40 years (18-86)*	Diabetes	Prevalence of medical risk factors -Diabetes mellitus: 10.52%; -Elevated blood glucose levels: 19.97% ^¥^
**Zhang, 2015 [****33****]**	China, upper-middle income	Longitudinal	415 starting ART (74%), 24 years (27.41) *^#^*	Diabetes	Incidence medical risk factors -Diabetes mellitus: 2.62 per 100 py -Impaired fasting glucose: 35.64 per 100 py
**Riyaten, 2015 [****34****]**	Thailand, upper-middle income	Longitudinal	1594 on ART (24%), *32.5 years (28.2-37.7) ^#^*	Diabetes	Incidence of medical risk factors -Diabetes mellitus: 5.0 per 1000 py (95%CI 3.8-6.6)
**Lo, 2009 [****35****]**	Taiwan, high income	Retrospective case-control	824 >95% on ART (92%), 34 years (28-40)**, of whom 50 new-onset DM cases; 100 matched controls >95% on ART (86%), 48 years (43-56)** without DM	Diabetes	Incidence of diabetes- -13.1 cases per 1000 person-years
**Srivanich 2010 [****36****]**	Thailand, Upper-middle income	Cross-sectional	149 non-diabetics 92% on ART (65%), 42.2 years (10.0)	Glucose levels	Prevalence of medical risk factors- -Impaired fasting glucose: 27.5%
**Hejazi, 2014 [****37****]**	Malaysia, upper-middle income	Cross-sectional	340 on ART: 185 normotensive (72%), *40.2 years (7.6)*; and 155 hypertensive (87%), *43.9 years (10.2)*	Hypertension	Prevalence medical risk factors -Hypertension: 45.6%
**Wu, 2012 [****38****]**	Taiwan, high income	Cross-sectional	803 on ART (95%), *metabolic syndrome: 44.5 years (9.7); and no metabolic syndrome: 36.8 years (10.6)*	Metabolic syndrome	Prevalence of medical risk factor -Metabolic syndrome: 26.2%
**Luo, 2014 [****39****]**	China, upper-middle income	Cross-sectional	455 on ART/ART naïve (66%), *38.1 years (8.8)*	Tobacco use	Prevalence of behavioural risk factors -Smoking: 61.7%
**Nguyen, 2015 [****40****]**	Vietnam, lower-middle income	Cross-sectional	1133 on ART (59%), *35.5 years (6.9)*	Tobacco use	Prevalence of behavioural risk factors -Current smoking: 36.1% -Past smoking: 9.5%
**Oka, 2013 [****41****]**	Japan, high income	Cross-sectional	100 on ART/ART naïve (100%), *42.1 years (12.7)*	Tobacco use	Prevalence behavioural risk factors -Smoking: 40%
**Lall, 2016 [****42****]**	India, lower-middle income	Cross-sectional	542 HIV+ (55%), 198208 HIV- (37%), *age unsp*.	Tobacco use	Prevalence behavioural risk factors: HIV+ *vs.* HIV- -Tobacco use (incl. smoking) in men: 68 *vs.* 58% (p<0.005) -Tobacco use (incl. smoking) in women: 12 *vs.* 11% (p=not tested) Differences between HIV+ and HIV- -Those with HIV more frequently were a smoker (OR 1.48, 95%CI 1.05-2.1, p<0.05)

Abbreviations: N=number; SD=standard deviation; ART=antiretroviral therapy; TB=tuberculosis; EFV=efavirenz; NVP=nevirapine; HIV+/-=HIV positive/negative individuals; unsp.=unspecified; OR=odds ratio; CI=confidence interval; p=probability; ns=not significant; *vs.*=versus; TC=total cholesterol; HDL=high density lipoprotein-cholesterol level; LDL=low density lipoprotein-cholesterol level; BP=blood pressure; py=person year. * Age in median (range); ^#^ age in median (IQR); ^^^ findings from multivariate analysis; ^¥^ *based on older definition of hyperglycaemia, >110 mg/dL*; *^±^ HDL <35 mg/dL; ° LDL>160 mg/dL; ~ TC >240 mg/dL; ^∞^LDL≥135 mg/dL; ^€^ Triglycerides>200 mg/dL.*

**Table 3:** CVD-related recommendations in national HIV guidelines from the Asian region.

**Country, year (refs)**	**Screening**	**Monitoring**	**Listing drug interactions**
**Korea, 2013 [****46****]**	Medical history Lipid profile Fasting blood sugar	Lipid profile Fasting blood sugar	No
**Philippines, 2009 [****47****]**	Lipid profile Fasting blood glucose	Lipid profile Fasting blood glucose	No
**Thailand, 2014 [****48****]**	Lipid profile Fasting blood glucose	Lipid profile Fasting blood glucose	No
**Malaysia, 2011 [****49****]**	Medical history Lipid profile Fasting blood glucose	Lipid profile Fasting blood glucose	Yes
**India, 2013 [****50****]**	Medical history Lipid profile Fasting blood glucose	Lipid profile Fasting blood glucose	Yes
**Vietnam, 2009 [****51****]**	Medical history Lipid profile Fasting blood glucose	Lipid profile Fasting blood glucose	Yes
**Cambodia, 2012 [****52****]**	Medical history Lipid profile Fasting blood glucose	Lipid profile	Yes
**Myanmar, 2011 [****53****]**	Not specified	Lipid profile Fasting blood glucose	Yes
**Hong Kong, 2013 [****54****]**	10-year CVD risk Medical history Family history of CVD Lipid profile Fasting blood glucose	10-year CVD risk Lipid profile Fasting blood glucose	Yes

English versions of guidelines were not available online for Japan, Indonesia, and Taiwan
